# Molecular analysis of immune checkpoint inhibitor associated erythema nodosum-like toxicity

**DOI:** 10.3389/fimmu.2025.1542499

**Published:** 2025-03-13

**Authors:** Xiaopeng Sun, Margaret L. Axelrod, Paula I. Gonzalez-Ericsson, Violeta Sanchez, Yu Wang, Jonathan L. Curry, Elizabeth J. Phillips, Yaomin Xu, Douglas B. Johnson, Justin M. Balko

**Affiliations:** ^1^ Department of Medicine, Vanderbilt University Medical Center, Nashville, TN, United States; ^2^ Department of Pathology and Immunology, Washington University in St. Louis, St. Louis, MO, United States; ^3^ Breast Cancer Research Program, Vanderbilt University Medical Center, Nashville, TN, United States; ^4^ Department of Biostatistics, Vanderbilt University Medical Center, Nashville, TN, United States; ^5^ Department of Pathology and Translational Molecular Pathology, The University of Texas MD Anderson Cancer Center, Houston, TX, United States

**Keywords:** immunotherapy, melanoma, irAE, erythema nodosum (EN), autoimmunity

## Abstract

**Purpose:**

Immune checkpoint inhibitors (ICIs) are increasingly used to treat advanced malignancy but can induce immune-related adverse events (irAE). The mechanisms behind these sporadic and sometimes life-threatening irAEs remain largely unexplored. Here, we present a case report and in-depth molecular analysis of an erythema nodosum (EN) like irAE occurring in a melanoma patient with isolated brain metastasis, aiming to explore the potential mechanism of this irAE.

**Methods:**

We performed RNA and T cell receptor (TCR) sequencing on the patient’s resected brain metastasis and biopsy of EN-like irAE. Single cell RNA/TCR sequencing was conducted on the patient’s peripheral blood mononuclear cells (PBMC) at baseline, 3 weeks after ipilimumab and nivolumab combination therapy, during EN toxicity and after resolution.

**Results:**

The site of EN-like irAE showed a distinct accumulation of pro-inflammatory immune cells, accompanied by the upregulation of inflammatory and interferon response signatures. In addition, clonal expansion and activation of irAE-associated CD8 T cells and upregulation of monocyte-specific interferon signatures occurred concurrently with irAE onset.

**Conclusion:**

The unique immune landscape at the EN-like irAE could indicate that this irAE is distinct from anti-tumor immune and analogous non-ICI autoimmune milieus. Our data also suggests that systemic immune activation induced by ICI treatment, as reflected in PBMC, may help monitor the patient’s treatment response and access irAE risk.

## Background

Immune checkpoint engagement is a key component in limiting autoimmune inflammation, maintaining fetal tolerance during pregnancy, and preventing the rejection of transplanted organs; however, it is also a common immune suppression mechanism that tumor cells can hijack to avoid immune surveillance. Immune checkpoint inhibitors (ICI), such as anti-PD-1/PD-L1 or anti-CTLA-4, can bind co-inhibitory immune checkpoint receptors and reactivate anti-tumor immunity. Currently, ICI has profoundly changed the treatment in 20 different cancer types, increasing response rate from 10-50% across various solid tumors (1, 2).

ICI can also cause immune-related adverse events (irAEs). Common ICI-induced irAEs include dermatitis, endocrinopathies, colitis, hepatitis, and pneumonitis, which are all thought to arise from aberrant activation of autoreactive T cells (3, 4). The rate of irAEs and severity vary by treatment regimen. From previous clinical experience in melanoma, CTLA-4 inhibition results in a high incidence of dose-dependent toxicities (high-grade toxicities in 38.6% and 57.9% of patients with metastatic melanoma receiving ipilimumab 3 mg/kg or 10 mg/kg, respectively) (5), while only 10-15% of patients receiving PD-1/PD-L1 experienced high-grade toxicity (6). Concurrent ICI use, such as combined anti-CTLA-4+anti-PD-1, also augments the risk of autoimmune toxicities, resulting in almost two-fold increased incidences of high-grade irAEs (7).

The molecular and cellular mechanisms of irAEs remain largely unclear, with a few limited studies on lichenoid and bullous pemphigoid irAEs (8, 9). To gain insights into these mechanisms, we describe a case of erythema nodosum (EN)-like irAE from a patient treated with combination ipilimumab and nivolumab and apply deep molecular analysis of the tumor, the EN-like toxicity, and longitudinal analysis of peripheral blood.

## Results

### Case report

**Figure 1:**
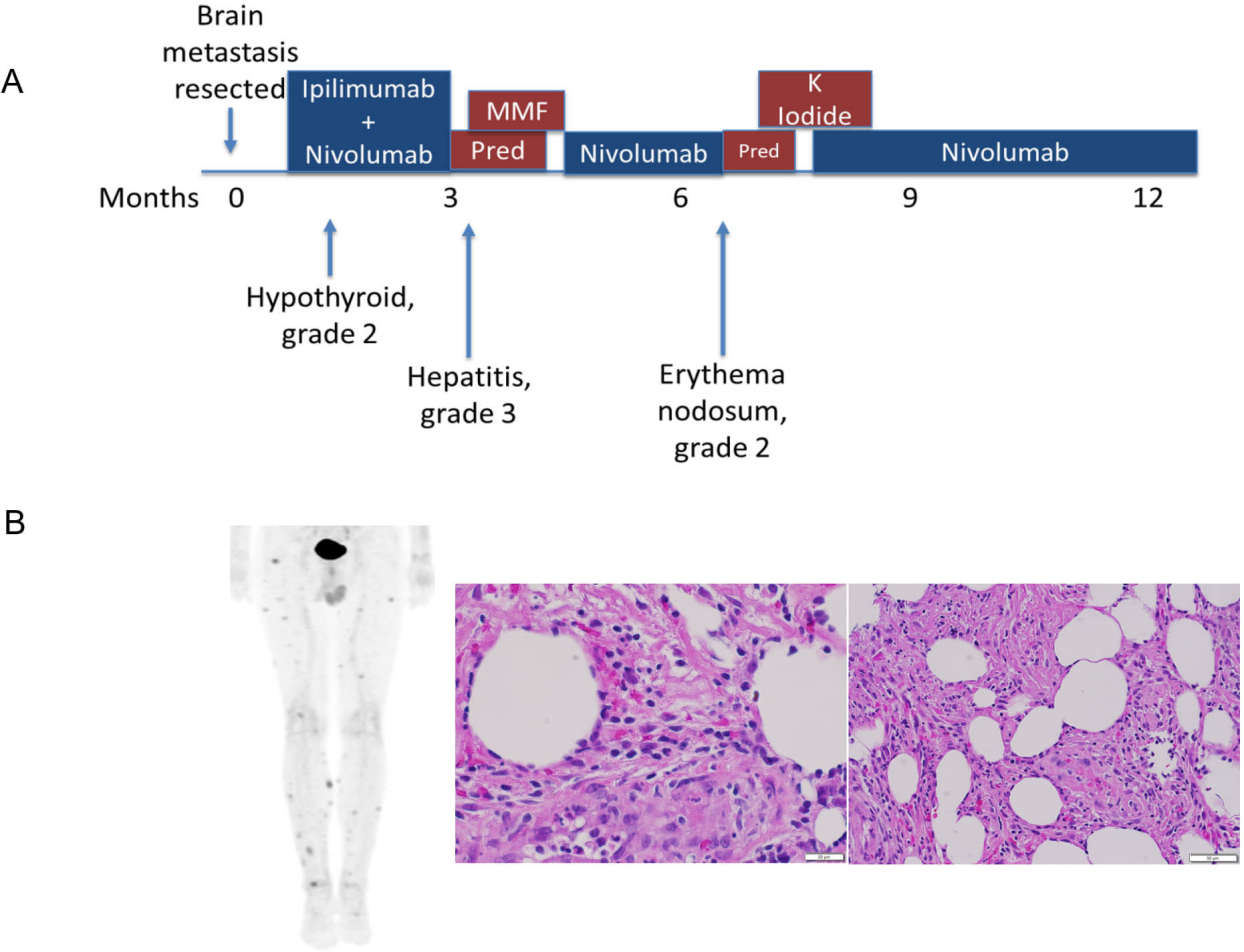
Clinical course of anti-PD-1-induced erythema nodosum. **(A)** The patient developed Erythema nodosum-like irAE approximated 6 months after metastasis resection and ICI treatment **(B)** PET-CT imaging demonstrating multiple FDG-avid subcutaneous nodules, which were biopsied and identified as erythema-nodosum-like panniculitis, as demonstrated by increased immune infiltration.

A man in his 40s without a history of known cutaneous melanoma presented with headaches and seizures and was found to have a hemorrhagic right parietal lobe mass. He underwent surgical resection which showed metastatic melanoma, and post-operative radiation therapy. Imaging showed no other intra- or extra-cranial disease. The patient received a combination of ipilimumab (anti-CTLA-4) and nivolumab (anti-PD-1) complicated by hypothyroidism and elevated liver enzymes after his second dose, which was treated by levothyroxine and prednisone, followed by mycophenolate mofetil, respectively. Following the resolution of liver enzymes, he received maintenance therapy with a single agent, nivolumab, without worsening liver function and with no evidence of a new metastatic disease (**Figure 1A**).

Approximately 4 months after resuming nivolumab, the patient developed an EN-like irAE. He developed painful subcutaneous nodules on his lower extremities and trunk. Although clinical pictures are not available for this patient, the irAE’s clinical presentation was similar to previously reported EN-like irAEs (10, 11). PET-CT showed multifocal FDG avid subcutaneous lesions concerning for metastatic disease. Biopsies of two different lesions both demonstrated adipose tissue with acute and chronic inflammation and panniculitis consistent with an EN-like reaction. IHC showed a mixture of CD4+ and CD8+ T cells, negative CD56, and rare CD20+ B cells (**Figure 1B**). The condition was effectively managed with potassium iodide treatment. Since EN-like irAE is not life-threatening toxicity and half of the patients experiencing irAEs do not have irAE recurrence on ICI rechallenge (12, 13), the nivolumab therapy was subsequently resumed, and the patient carefully monitored. The patient stopped therapy after approximately two years and has had no additional recurrences of tumor or irAEs approximately 5.5 years after the initial presentation. As EN is a rarely reported irAE (10, 11, 14, 15), we sought to elucidate the pathogenesis by conducting an extensive examination of samples from the patient’s blood, resected brain metastasis, and a tissue biopsy from the EN toxicity site.

### Profound immune cell infiltration and immune activation in EN nodules

We performed bulk RNA sequencing on the patient’s irAE biopsy, resected brain metastasis, and four cases of non-ICI-related skin autoimmune disease samples (three EN and one granulomatous disease [GD]) as non-ICI-induced skin condition comparators.

**Figure 2:**
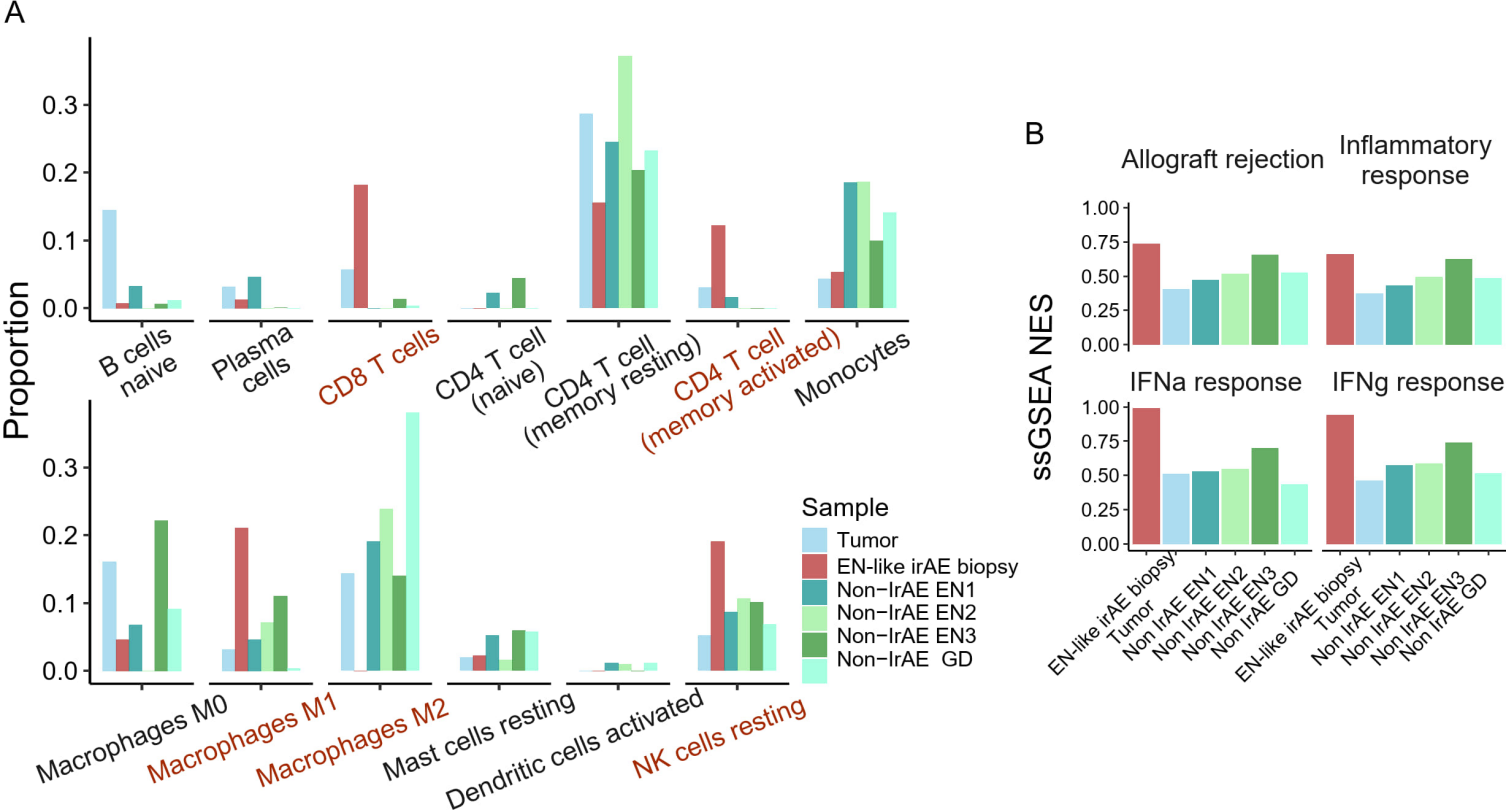
Site of ICI-induced erythema nodosum harbors pro-inflammatory immune cells. **(A)** CibersortX immune cell deconvolution using LM22 reference of the site of toxicity, tumor, and non-ICI induced skin autoimmune disease. **(B)** Enrichment score of Hallmark immune-related pathways.

Compared to all other samples, the EN-like irAE demonstrated enrichment of pro-inflammatory leukocytes RNA signatures, including CD8+ T cells, memory-activated CD4+ T cells, M1 macrophages, and resting NK cells (**Figure 2A**). Notably, immunosuppressive M2 macrophages, which were abundant in tumor and non-ICI related EN, were nearly undetectable in the EN-like irAE (**Figure 2A**). Furthermore, we observed enrichment of immune activation signatures at the toxicity site, evidenced by elevated enrichment scores for Hallmark pathways “inflammatory response”, “interferon response”, and “allograft rejection” when compared to tumor and non-ICI autoimmune skin disorders (**Figure 2B**). Since the response to anti-PD-1 therapy has been linked to type-II interferon responses (16, 17), these findings suggest that the toxicity site is characterized by an ICI-associated immune activation pattern reminiscent of anti-tumor immune responses.

### Distinct TCR clonal expansion patterns in toxicity sites and peripheral blood

**Figure 3:**
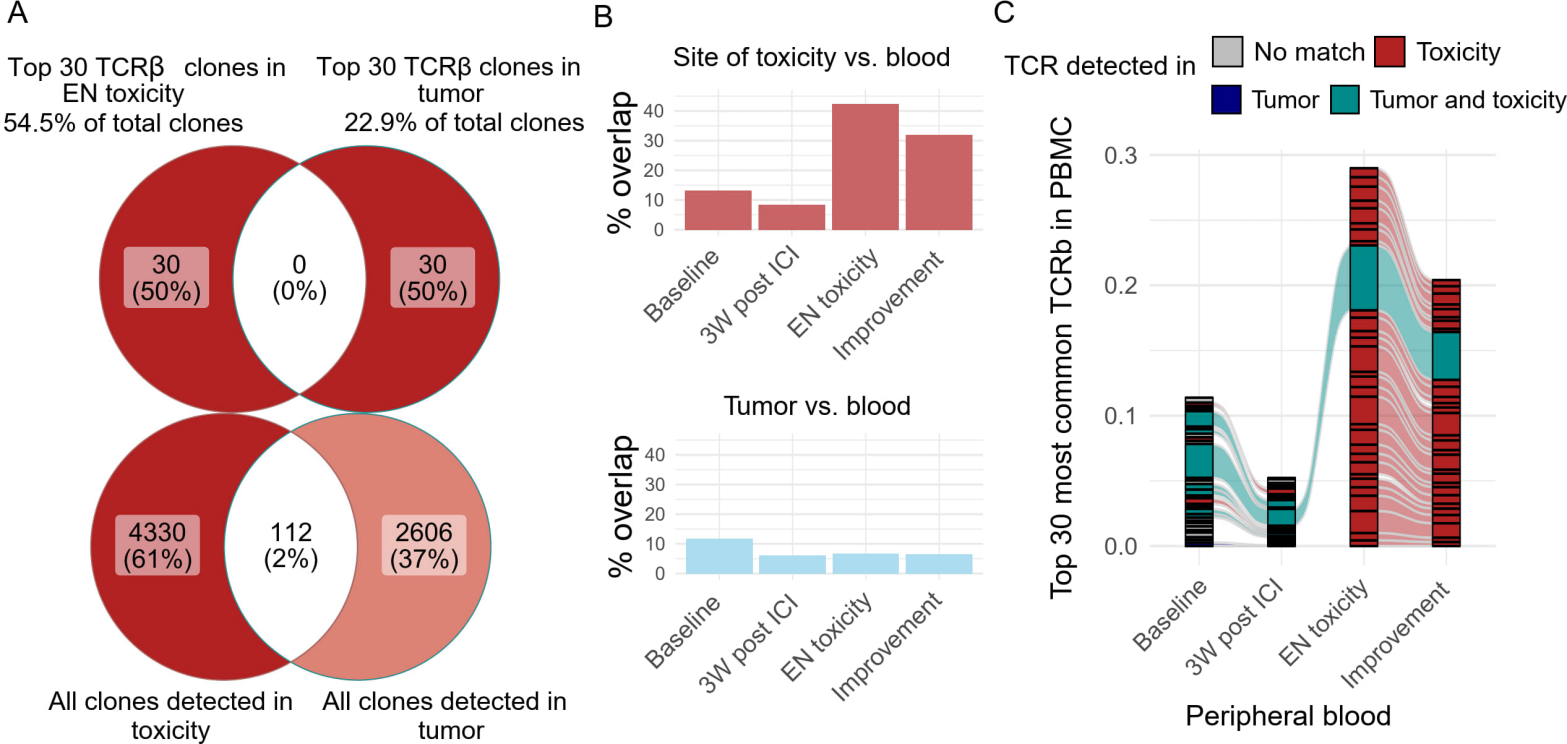
Toxicity-associated TCRs were enriched in peripheral blood. **(A)** Venn diagram depicting overlapping TCR beta clones between the site of toxicity and the patient’s original tumor mass. **(B)** The percentage of peripheral blood TCRb that overlaps with clones detected on the site of toxicity(red) or tumor(blue). **(C)** Flow diagram of the top 30 most abundant TCRb clones detected in the longitudinal blood samples, colored by the site where the clone was detected.

One proposed mechanism for ICI-induced irAE is that the site of toxicity and the tumor share a common antigen(s), leading to T cells indiscriminately attacking healthy tissue upon the loss of negative modulation by immune checkpoints (18). To test whether the EN-like irAE and the patient’s original tumor harbored T cells that shared similar T cell repertoire, which could support this hypothesis, we extracted and compared T cell receptor (TCR) -beta sequences across these sites. There was minimal overlap in TCR clones between these sites (**Figure 3A**). Given that the brain metastasis was removed nearly six months before the onset of EN toxicity, it remains possible that the T cells had experienced clonal evolution during ICI treatment, resulting in novel TCRs.

Previous research demonstrated that clonal expansion of peripheral T cells is associated with the development of irAE (19). Next, we tested whether toxicity-associated TCRs could be detected in the peripheral blood and if they expanded during ICI-EN. TCR-beta sequences were extracted from longitudinal peripheral blood samples collected post-brain metastasis removal (Baseline), 3 weeks post-ICI (3W post-ICI), during EN toxicity, and after symptom improvement (Improvement). Overall, 11.6% of TCR-beta clonotypes in the baseline blood sample overlapped with the tumor. The proportion of tumor-overlapping clonotypes decreased post-surgical resection and with the initiation of ICI (**Figure 3B**). During EN toxicity, 42% of the clonotypes detected in the blood overlapped with those at the toxicity site. The proportion of overlapping clones slightly decreased after symptom improvement (**Figure 3B**). Additionally, we observed clear clonal replacement in the blood, with previously undetected, toxicity-associated clones becoming heavily enriched during the onset of toxicity (**Figure 3C**). Overall, the TCR data suggests that the systemic clonal dynamics in the blood may reflect the onset and resolution of irAE.

### Systemic immune dynamics during irAE onset

**Figure 4:**
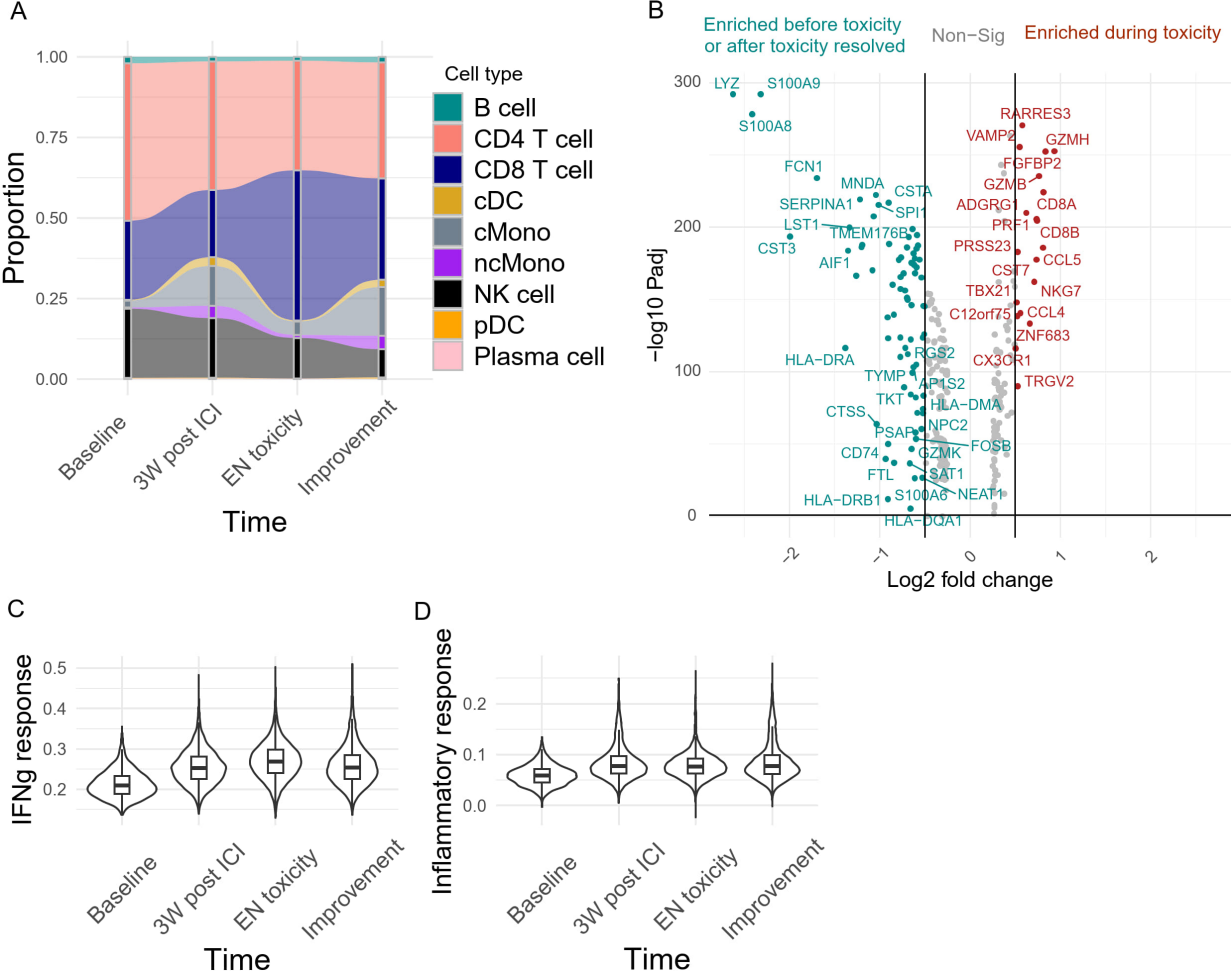
Systemic immune dynamics during irAE onset **(A)** Peripheral blood immune cell abundance monitored longitudinally. **(B)** Differential gene expression analysis of the peripheral blood capturing genes upregulated during EN toxicity (right, red) and pre/post-toxicity (left, green). Adjusted P value <0.01 and absolute log2 fold change >0.5. The per-cell enrichment score of **(C)** Hallmark interferon gamma response, and **(D)** Inflammatory response pathway across 4 different time points in the peripheral blood.

To further characterize the changes in systemic immunity during irAE onset, we performed single-cell RNA/TCR sequencing on the patient’s longitudinal PBMC samples. We observed a decrease in peripheral classical monocyte (cMono) abundance concurrent with an expansion of CD8+ T cells during EN-like irAE (**Figure 4A**). Differential gene expression analysis revealed a downregulation of myeloid signature genes, such as *LYZ*, *S100A8*, and *S100A9*, during EN-like irAE compared to pre- and post-toxicity timepoints (**Figure 4B**). In contrast, genes associated with CD8+ T cell cytotoxicity, such as *GZMB, NKG7, and PRF1*, were upregulated during EN-like irAE (**Figure 4B**). Additionally, our ssGSEA analysis showed an upregulation of interferon response and inflammatory response-related genes post-ICI treatment, indicating a systemic immune response induced by ICI (**Figures 4C, D**).

**Figure 5:**
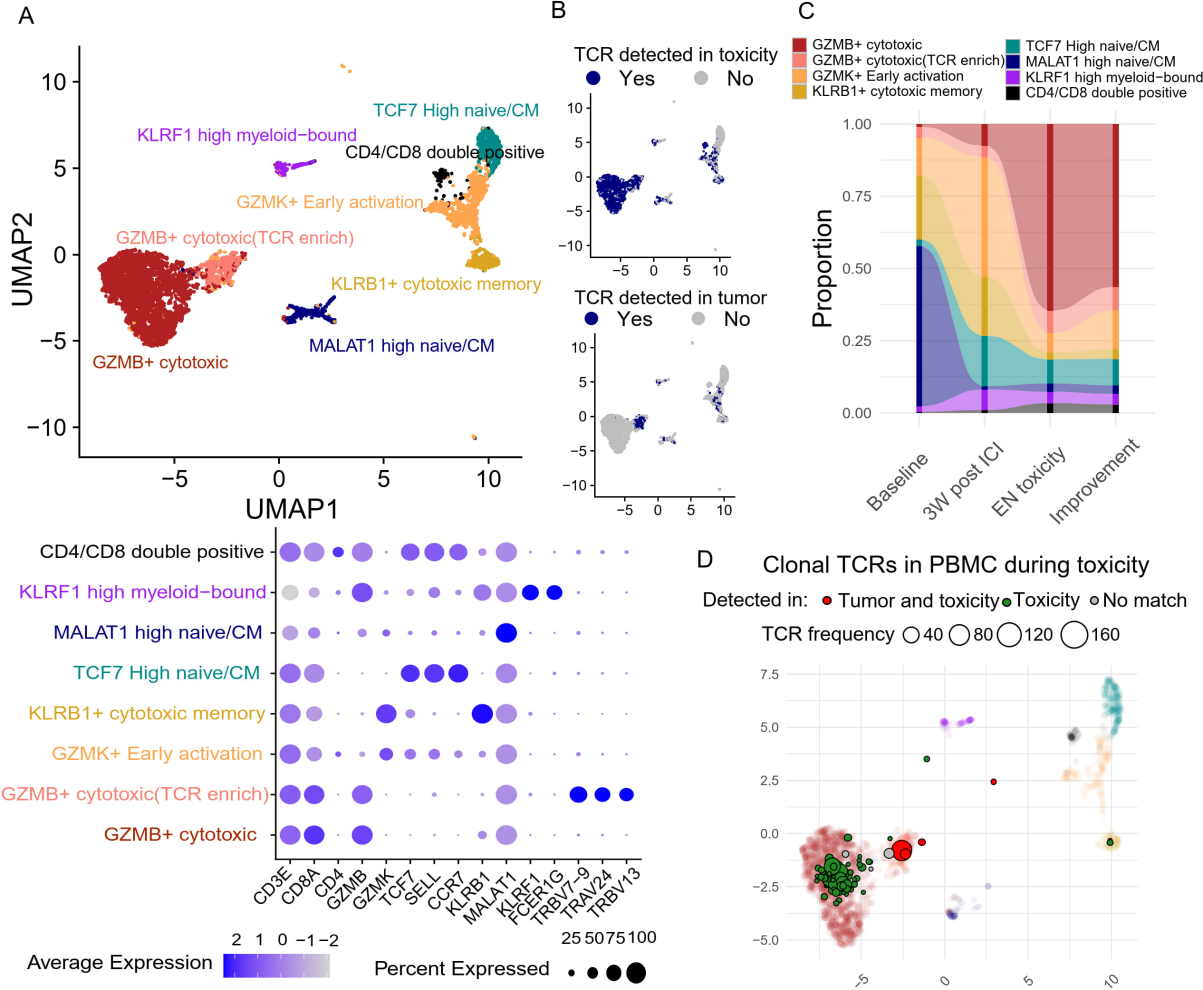
Peripheral blood CD8+ T cell dynamics and their association with tumor and site of toxicity based on TCR clone overlap **(A)** Unsupervised clustering based on transcriptomic features generates eight distinct CD8 T cell clusters, with their corresponding gene features presented in the dot plot. **(B)** TCR clones of each T cell are compared against tumor and site of toxicity to identify overlapping clones. **(C)** The abundance of each CD8 T cell cluster was tracked longitudinally. **(D)** The center location of each clonal (>3 T cell sharing same TCR) TCR clones on the Umap during toxicity onset. Toxicity-overlapping TCRs are colored green, and tumor+toxicity-overlapping TCRs are colored red.

Sub-clustering of the CD8+ T cells resulted in eight functionally distinct clusters, including naïve/central memory, newly activated, cytotoxic, and effector memory T cells (**Figure 5A**). There was a dominant population of GZMB+ cytotoxic CD8 T cells, carrying TCRs that were also detected at the site of EN-like irAE (**Figure 5B**), alongside a smaller subset that shared nearly identical functional markers but was uniquely enriched for specific TRBV and TRAV sequences; these T cells had TCRs that were detected in both the tumor and EN-like irAE biopsy (**Figure 5B**). Longitudinal analysis of CD8+ T cells revealed that ICI treatment induced a significant expansion of GZMK+ early activated CD8+ T cells (**Figure 5C**), potentially reflecting early T cell priming. As EN toxicity developed, GZMB+ cytotoxic CD8+ T cells became the dominant phenotype (**Figure 5C**), indicating peripheral CD8+ T cell activation and potential clonal expansion. Furthermore, during EN toxicity, most clonal T cells in the peripheral blood carrying EN associated TCRs were the activated, GZMB+ cytotoxic subtype (**Figure 5D**). This suggests that the clonal expansion and activation of CD8+ T cells, particularly the GZMB+ cytotoxic subtype, are closely associated with the development of EN toxicity.

**Figure 6:**
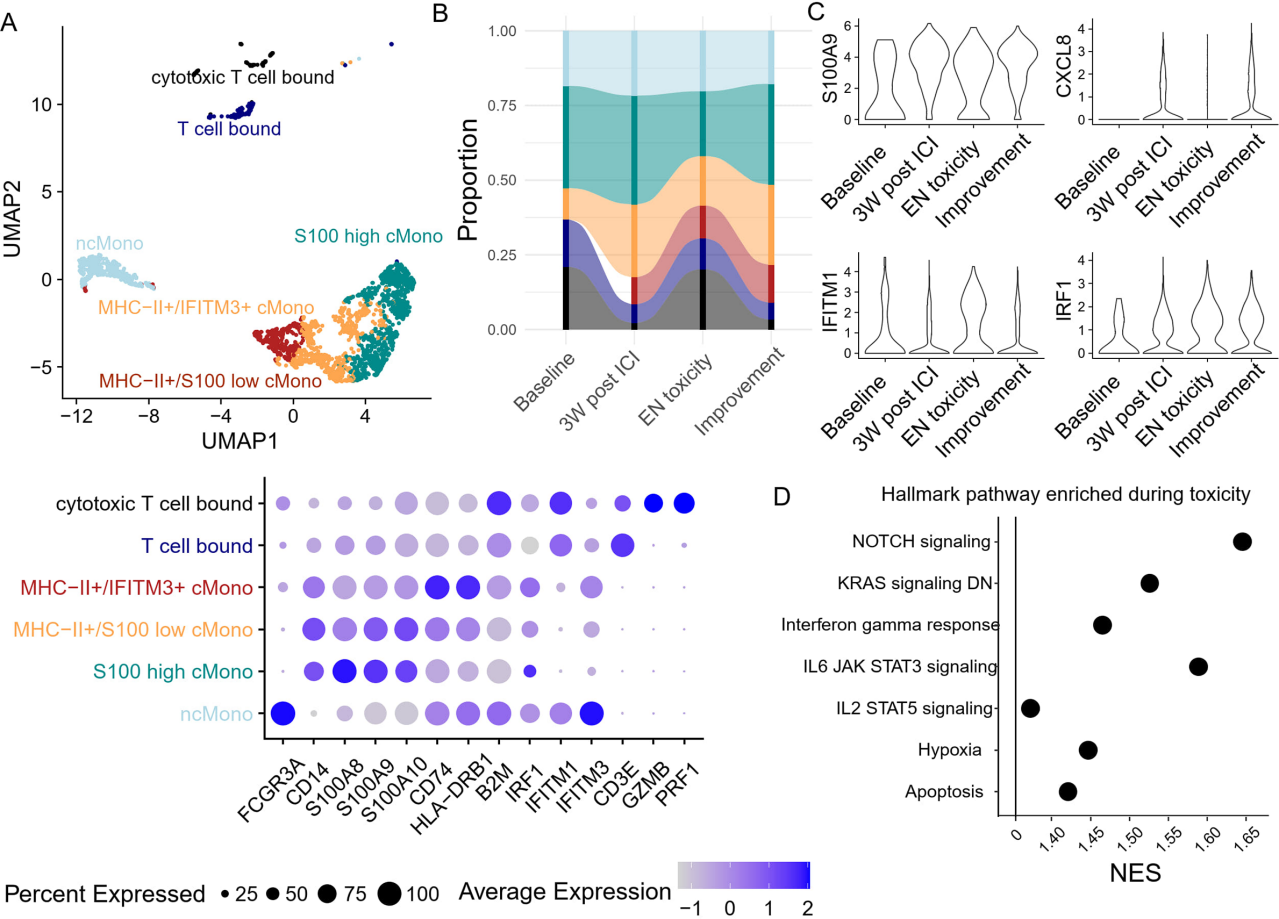
Peripheral blood monocyte dynamics and transcriptomic changes during toxicity onset **(A)** Unsupervised clustering based on transcriptomic features generates six monocyte clusters, with their corresponding gene features in the dot plot. **(B)** The abundance of each monocyte cluster was tracked longitudinally. **(C)** The expression of S100A9, CXCL8, and two interferon response-related genes in monocytes was monitored longitudinally. **(D)** Gene set enrichment analysis based on the differentially expressed genes between toxicity onset vs. pre/post toxicity. Only pathways with adjusted p-values <0.05 are presented.

Since the proportion of cMono decreased after EN toxicity, we decided to further investigate whether the development of EN-like irAEs also coincides with a change in monocyte phenotype. Monocytes were further divided into six clusters based on their transcriptomic features, specifically by the expression of MHC-II transcripts, S100s, and interferon response genes (**Figure 6A**). Two clusters also expressed T cell-associated genes, which may represent monocyte-T cell doublets or physiological interacting cells. Apart from the increase in T cell-bound monocytes during toxicity, no significant changes were observed in other monocyte subclusters (**Figure 6B**). However, differential gene expression analysis revealed a reduction in S100 gene expression, which was previously reported to be a marker of immunosuppressive phenotypes (20, 21), and a concurrent increase in interferon response element expression (**Figure 6C**). Further GSEA analysis supported the observation of increased interferon and cytokine responses during EN toxicity (**Figure 6D**). These findings suggest a shift in the monocyte landscape towards a more pro-inflammatory state, potentially contributing to the pathogenesis of ICI-induced EN.

## Discussion

The mechanism behind ICI-induced irAEs remains largely case-specific. While theoretically, these toxicities can affect nearly every organ, dermatologic cases are among the most common irAEs for ICI-treated patients (22, 23). Here, we report our observations on the clinical and molecular features of ICI-induced EN in a melanoma patient. A detailed description of the patient’s peripheral immune signature during irAE highlights the role of systemic immunity during this rare cutaneous toxicity.

Several cases of EN-like irAE have been reported in ICI treated melanoma patients, with isolated cases in clear cell carcinoma, esophageal cancer, and renal cell carcinoma (10, 11, 14, 15). irAE onset ranges from 4 weeks to a year after ICI treatment. Among most cases, increased lymphocytes, histiocytes, and neutrophils were observed with no sign of infection, similar to the immune infiltration pattern observed in our case (10). In addition, prior or concomitant hypothyroidism was found in two EN toxicity cases (10), hinting at a potential association between these conditions. Although sparse HLA typing data were available for prior reported cases, upon comparing the patients’ HLA typing, our case shared HLA-B*35 and HLA-DPB1*04, two frequently carried alleles, with a previously reported EN-like irAE patient who had a prior medical history of hypothyroidism (10), suggesting the association between HLA genotype and the toxicity pathogenesis.

The basic cancer immunity cycle suggests that antigens released from tumors during cell turnover and in response to therapies drain to the lymph node, where they are acquired by professional antigen presenting cells and presented to T lymphocytes for priming. Appropriately primed T cells are ‘licensed’ to leave the lymph node and seek out sources of inflammation, whereby they must pass through the systemic peripheral circulation as a conduit (24). Similarly, irAE induced by bystander effects from activated T-cells or T cells targeting healthy organ-tumor overlapping antigens could use a similar adaptive immune response cycle (19, 25). Previous studies have shown that peripheral activated CD4 memory T cell abundance and TCR diversity are strongly associated with irAE onset (25). Using longitudinal peripheral blood, we showed that expansion of cytotoxic CD8 T cells and increased monocytic interferon response were also strongly associated with EN toxicity onset. Together, those data suggest that systemic immune activation induced by ICI treatment may also reflect the risk of irAE, providing a potential method to monitor the patient’s treatment response and irAE risk assessment. However, these results also suggest that differential mechanisms (for example dominated by CD4 or CD8 T cell responses) may exist among irAEs.

One popular mechanistic explanation of irAE is that shared antigens between the tumor and the affected organ may cause a break in self-tolerance during anti-tumor immune responses (18, 26). In this unique case, however, the TCRs detected were not shared between the site of irAE and the original brain metastasis that was resected 6 months prior. It is still possible that the TCRs we detected in the site of toxicity may target potential micrometastases that we were not able to detect and/or sample. It is important to note that the development of irAEs has been correlated with anti-tumor immunity and response to ICI (27–29). The T cell activation signatures observed in the peripheral blood support the idea that ICI could induce long-term constant immune surveillance against cancer, even when the patient appears to be disease free.

Previous analysis demonstrated that clonal T cell activation in tumors and peripheral blood is associated with better ICI treatment response in melanoma. As demonstrated by our data and other studies (19, 25, 30, 31), systemic T cell expansion is also associated with the development of irAE. Currently, one of the challenges of irAE management is early detection and biomarker development. Using peripheral biomarkers such as T cell clonality and T cell activation, it may be possible to detect patients experiencing systemic immune responses and enhance patient monitoring to mitigate potential severe irAEs. Nonetheless, further research is needed to determine whether such markers can be used to monitor patients for development of potential irAEs.

In conclusion, we present a deep molecular analysis of ICI-induced EN. In addition, the observation of systemic inflammation during toxicity onset further strengthens the importance of systemic immunity during ICI and the development of irAEs.

## Methods

### Patient information

The patient was treated at Vanderbilt University Medical Center and consented to the clinical and biospecimen repository (IRB#100178). Peripheral blood samples were taken at baseline, early on treatment, and at time of toxicity per our protocol. FFPE samples from resected brain metastasis and skin biopsies were obtained from pathology. Samples from patients non-ICI autoimmune disorders were obtained from pathology (IRB# 150754).

### RNA sequencing and data analysis

Total RNA was isolated from FFPE tumor, ICI-EN and non-ICI skin autoimmune disease biopsy samples using the Promega Maxwell 16 FFPE RNA kits per the manufacturer’s protocol. mRNA enrichment and cDNA library were prepared utilizing the stranded mRNA (polyA-selected) library preparation kit. Sequencing was performed at Paired-End 150 bp on the Illumina NovaSeq 6000 targeting an average of 50M reads per sample. Demultiplexed FASTQ files were next aligned using STAR with a genome index generated from human Hg38. FeatureCount was next applied to create gene count matrix. Subsequent MultiQC was performed to ensure sample homogeneity. Raw count generated by FeatureCount was imported to R. Genes that were expressed in less than 50% of the samples were excluded from the analysis. The filtered gene count matrix was next used to generate DESeq2 objects with corresponding metadata. Raw gene counts were transformed using VST transformation followed by GSVA enrichment analysis which assigns enrichment scores of the Hallmark pathways to each sample. The transformed gene sets were deconvoluted with CIBERSORTx using the LM22 matrix to obtain immune cell composition in each biopsy.

### TCR sequencing

Using the whole RNA extracted from FFPE biopsies, TCRs were sequenced using the TCR Immunoverse all chain assay per the manufacturer’s protocol (Invitae/ArcherDX). Sequencing results were evaluated using Archer Immunoverse analyser. CDR3 sequences and frequency tables were extracted from the manufacturers’ analysis platform. TCR beta sequence was extracted to identify matching clones among tumor biopsy, ICI-EN site, and peripheral blood.

### Single cell RNA sequencing and data analysis

Each sample (targeting 5,000–15,000 cells per sample) was processed for single-cell 5′ RNA and TCR sequencing utilizing the 10x Chromium system. Libraries were prepared following the manufacturer’s protocol. The libraries were sequenced using NovaSeq 6000 with 150 bp paired-end reads. RTA (v.2.4.11; Illumina) was used for base calling, and analysis was completed using 10x Genomics Cell Ranger software. Data were analyzed in R using the filtered h5 gene matrices in the Seurat package (32). In brief, samples were subset to include cells with >200 but <3,000 unique transcripts to exclude probable non-cellular RNA reads and doublets. Cells with >15% of reads coming from mitochondrial transcripts were also excluded as probable dying cells. General immune cell subtypes were imputed using *scPred* (33). CD8 T cell clusters were generated with *FindClusters* function after removing all CD8 T cell without TCR information. Monocyte clusters were generated in by setting FindClusters. Detailed cell subtype identify was given based on top10 differentially expressed genes in each cluster.

Differential gene expression analysis was performed across all cells using FindMarkers function between different timepoints. Gene set enrichment analysis was performed to obtain pathway enrichment score by taking the log2 fold change and p-value from differential gene expression results. Besides pair-wise comparison, enrichment scores for each Hallmark pathways is generated for each cell using Escape (34).

### HLA typing

We performed four-digit class I and II typing of HLA (with Illumina MiSeq) for the HLA antigens ABC, DR, DQ, and DP on DNA extracted from peripheral blood.

## Data Availability

The original contributions presented in the study are included in the article/supplementary files, further inquiries can be directed to the corresponding author/s.

## References

[1] Yarchoan M Hopkins A Jaffee EM. Tumor mutational burden and response rate to PD-1 inhibition. N Engl J Med. (2017) 377:2500–1. doi: 10.1056/NEJMc1713444 PMC6549688 29262275

[2] Zhao B Zhao H Zhao J. Efficacy of PD-1/PD-L1 blockade monotherapy in clinical trials. Ther Adv Med Oncol. (2020) 12:1758835920937612. doi: 10.1177/1758835920937612 32728392 PMC7366397

[3] Postow MA Sidlow R Hellmann MD. Immune-related adverse events associated with immune checkpoint blockade. New Engl J Med. (2018) 378:158–68. doi: 10.1056/NEJMra1703481 29320654

[4] Johnson DB Chandra S Sosman JA. Immune checkpoint inhibitor toxicity in 2018. JAMA. (2018) 320:1702–3. doi: 10.1001/jama.2018.13995 30286224

[5] Tarhini AA Lee SJ Hodi FS Rao UNM Cohen GI Hamid O. Phase III study of adjuvant ipilimumab (3 or 10 mg/kg) versus high-dose interferon alfa-2b for resected high-risk melanoma: north american intergroup E1609. J Clin Oncol. (2020) 38:567–75. doi: 10.1200/JCO.19.01381 PMC7030886 31880964

[6] Weber J Mandala M Vecchio MD Gogas HJ Arance AM Cowey CL. Adjuvant nivolumab versus ipilimumab in resected stage III or IV melanoma. New Engl J Med. (2017) 377:1824–35. doi: 10.1056/NEJMoa1709030 28891423

[7] Wolchok JD Chiarion-Sileni V Gonzalez R Rutkowski P Grob J-J Cowey CL. Overall survival with combined nivolumab and ipilimumab in advanced melanoma. N Engl J Med. (2017) 377:1345–56. doi: 10.1056/NEJMoa1709684 PMC5706778 28889792

[8] Curry JL Reuben A Szczepaniak-Sloane R Ning J Milton DR Lee CH. Gene expression profiling of lichenoid dermatitis immune-related adverse event from immune checkpoint inhibitors reveals increased CD14+ and CD16+ monocytes driving an innate immune response. J Cutaneous Pathol. (2019) 46:627–36. doi: 10.1111/cup.13454 30883858

[9] Marques-Piubelli ML Seervai RNH Mudaliar KM Ma W Milton DR Wang J. Gene expression profiling and multiplex immunofluorescence analysis of bullous pemphigoid immune-related adverse event reveal upregulation of toll-like receptor 4/complement-induced innate immune response and increased density of T1 T-cells. J Cutaneous Pathol. (2023) 50:661–73. doi: 10.1111/cup.14442 37150813

[10] Tetzlaff MT Jazaeri AA Torres-Cabala CA Korivi BR Landon GA Nagarajan P. Erythema nodosum-like panniculitis mimicking disease recurrence: A novel toxicity from immune checkpoint blockade therapy—Report of 2 patients. J Cutaneous Pathol. (2017) 44:1080–6. doi: 10.1111/cup.2017.44.issue-12 28901560

[11] Pach J Moody K Ring N Panse G Zhang M Deverapalli S. Erythema nodosum-like panniculitis associated with immune checkpoint inhibitor therapy: Two cases reporting a rare cutaneous adverse event. JAAD Case Rep. (2021) 13:118. doi: 10.1016/j.jdcr.2021.05.002 34189226 PMC8220292

[12] Dolladille C Ederhy S Sassier M Cautela J Thuny F Cohen AA. Immune checkpoint inhibitor rechallenge after immune-related adverse events in patients with cancer. JAMA Oncol. (2020) 6:865–71. doi: 10.1001/jamaoncol.2020.0726 PMC7163782 32297899

[13] Pollack MH Betof A Dearden H Rapazzo K Valentine I Brohl AS. Safety of resuming anti-PD-1 in patients with immune-related adverse events (irAEs) during combined anti-CTLA-4 and anti-PD1 in metastatic melanoma. Ann Oncol. (2018) 29:250–5. doi: 10.1093/annonc/mdx642 PMC5834131 29045547

[14] Choi ME Lee KH Won CH Chang SE Lee MW Choi JH. A case of erythema nodosum-like panniculitis induced by nivolumab in a patient with oesophageal cancer. Australas J Dermatol. (2019) 60:154–6. doi: 10.1111/ajd.2019.60.issue-2 30656640

[15] Seban R-D Vermersch C Champion L Bonsang B Roger A Ghidaglia J. Immune-related erythema nodosum mimicking in transit melanoma metastasis on [18F]-FDG PET/CT. Diagnostics. (2021) 11:747. doi: 10.3390/diagnostics11050747 33922013 PMC8143543

[16] Grasso CS Tsoi J Onyshchenko M Abril-Rodriguez G Ross-Macdonald P Wind-Rotolo M. Conserved interferon-γ Signaling drives clinical response to immune checkpoint blockade therapy in melanoma. Cancer Cell. (2020) 38:500–515.e3. doi: 10.1016/j.ccell.2020.08.005 32916126 PMC7872287

[17] Benci JL Johnson LR Choa R Xu Y Qiu J Zhou Z. Opposing functions of interferon coordinate adaptive and innate immune responses to cancer immune checkpoint blockade. Cell. (2019) 178:933–948.e14. doi: 10.1016/j.cell.2019.07.019 31398344 PMC6830508

[18] Johnson DB Balko JM Compton ML Chalkias S Gorham J Xu Y. Fulminant myocarditis with combination immune checkpoint blockade. N Engl J Med. (2016) 375:1749–55. doi: 10.1056/NEJMoa1609214 PMC5247797 27806233

[19] Subudhi SK Aparicio A Gao J Zurita AJ Araujo JC Logothetis CJ. Clonal expansion of CD8 T cells in the systemic circulation precedes development of ipilimumab-induced toxicities. Proc Natl Acad Sci. (2016) 113:11919–24. doi: 10.1073/pnas.1611421113 PMC5081579 27698113

[20] Zhao F Hoechst B Duffy A Gamrekelashvili J Fioravanti S Manns MP. S100A9 a new marker for monocytic human myeloid-derived suppressor cells. Immunology. (2012) 136:176–83. doi: 10.1111/j.1365-2567.2012.03566.x PMC3403264 22304731

[21] Hao W Zhang Y Dou J Cui P Zhu J. S100P as a potential biomarker for immunosuppressive microenvironment in pancreatic cancer: a bioinformatics analysis and. Vitro study. BMC Cancer. (2023) 23:997. doi: 10.1186/s12885-023-11490-1 37853345 PMC10585823

[22] Xu C Chen Y-P Du X-J Liu J-Q Huang C-L Chen L. Comparative safety of immune checkpoint inhibitors in cancer: systematic review and network meta-analysis. BMJ. (2018) 363:k4226. doi: 10.1136/bmj.k4226 30409774 PMC6222274

[23] Arnaud-Coffin P Maillet D Gan HK Stelmes J-J You B Dalle S. A systematic review of adverse events in randomized trials assessing immune checkpoint inhibitors. Int J Cancer. (2019) 145:639–48. doi: 10.1002/ijc.v145.3 30653255

[24] Hiam-Galvez KJ Allen BM Spitzer MH. Systemic immunity in cancer. Nat Rev Cancer. (2021) 21:345–59. doi: 10.1038/s41568-021-00347-z PMC8034277 33837297

[25] Lozano AX Chaudhuri AA Nene A Bacchiocchi A Earland N Vesely MD. T cell characteristics associated with toxicity to immune checkpoint blockade in patients with melanoma. Nat Med. (2022) 28:353–62. doi: 10.1038/s41591-021-01623-z PMC8866214 35027754

[26] Berner F Bomze D Diem S Ali OH Fässler M Ring S. Association of checkpoint inhibitor–induced toxic effects with shared cancer and tissue antigens in non–small cell lung cancer. JAMA Oncol. (2019) 5:1043–7. doi: 10.1001/jamaoncol.2019.0402 PMC6487908 31021392

[27] Eggermont AMM Kicinski M Blank CU Mandala M Long GV Atkinson V. Association between immune-related adverse events and recurrence-free survival among patients with stage III melanoma randomized to receive pembrolizumab or placebo: A secondary analysis of a randomized clinical trial. JAMA Oncol. (2020) 6:519–27. doi: 10.1001/jamaoncol.2019.5570 PMC6990933 31895407

[28] Maher VE Fernandes LL Weinstock C Tang S Agarwal S Brave M. Analysis of the association between adverse events and outcome in patients receiving a programmed death protein 1 or programmed death ligand 1 antibody. J Clin Oncol. (2019) 37:2730–7. doi: 10.1200/JCO.19.00318 31116675

[29] Das S Johnson DB. Immune-related adverse events and anti-tumor efficacy of immune checkpoint inhibitors. J Immunother Cancer. (2019) 7:306. doi: 10.1186/s40425-019-0805-8 31730012 PMC6858629

[30] Ostmeyer J Park JY Itzstein MS von Hsiehchen D Fattah F Gwin M. T-cell tolerant fraction as a predictor of immune-related adverse events. J Immunother Cancer. (2023) 11:e006437. doi: 10.1136/jitc-2022-006437 37580069 PMC10432621

[31] Kim KH Hur JY Cho J Ku BM Koh J Koh JY. Immune-related adverse events are clustered into distinct subtypes by T-cell profiling before and early after anti-PD-1 treatment. Oncoimmunology. (2020) 9:1722023. doi: 10.1080/2162402X.2020.1722023 32076579 PMC6999841

[32] Stuart T Butler A Hoffman P Hafemeister C Papalexi E Mauck WM. Comprehensive integration of single-cell data. Cell. (2019) 177:1888–1902.e21. doi: 10.1016/j.cell.2019.05.031 31178118 PMC6687398

[33] Alquicira-Hernandez J Sathe A Ji HP Nguyen Q Powell JE. scPred: accurate supervised method for cell-type classification from single-cell RNA-seq data. Genome Biol. (2019) 20:264. doi: 10.1186/s13059-019-1862-5 31829268 PMC6907144

[34] Borcherding N Vishwakarma A Voigt AP Bellizzi A Kaplan J Nepple K. Mapping the immune environment in clear cell renal carcinoma by single-cell genomics. Commun Biol. (2021) 4:1–11. doi: 10.1038/s42003-020-01625-6 33504936 PMC7840906

